# Correction: Postnatal infection surveillance by telephone in Dar es Salaam, Tanzania: An observational cohort study

**DOI:** 10.1371/journal.pone.0287801

**Published:** 2023-06-23

**Authors:** Susannah L. Woodd, Abdunoor M. Kabanywanyi, Andrea M. Rehman, Oona M. R. Campbell, Asila Kagambo, Warda Martiasi, Louise T. Day, Alexander M. Aiken, Wendy J. Graham

The seventh author’s name is spelled incorrectly. The correct name is: Louise T. Day.
