# Supplementary material for: Postnatal infection surveillance by telephone in Dar es Salaam, Tanzania: An observational cohort study
Source: PLoS One. 2021 Jul 1;16(7):e0254131. doi: 10.1371/journal.pone.0254131 (PMC8248639; doi:10.1371/journal.pone.0254131)
Supplement: S1 Appendix — (DOCX) [file pone.0254131.s001.docx]

**S1 Appendix: Telephone Questionnaire – Day 28**

Patient ID Number:_____________

**Information collected previously from hospital records and last interview:**

Delivery mode:

1. Vaginal delivery
2. Caesarean section

Baby alive at last phone survey? Y/N

Number of babies:________

**Introduction**

I am ____________________ from Ifakara Health Institute, phoning to ask questions as part of the CLEAN study.

I would like to ask you about your health since we last spoke 3 weeks ago.

Is this a convenient time to speak? (If No, arrange another time to call back)

Are you happy to continue with the survey? Y/N If Yes, continue with maternal questions

If No, are you happy for the answers from 3 weeks ago and the information from your medical records to still be used in the research? Y/N

Thank her for her time and end.

**Maternal questions**

Firstly, I will ask some questions about your health:

1. Have you been unwell or suffered any problems/complications since we phoned you 3 weeks ago? Y/N If No -> Qu.3
2. If Yes, Can you describe the problem and any diagnosis given (free-text).

______________________________________________________________________

1. For each of the following symptoms, can you tell me if you have experienced it in the last 3 weeks (since we phoned) and if you are still experiencing it today?

|  | Symptom | Last 3 weeks Y/N | Today Y/N |
| --- | --- | --- | --- |
| a. | Fever |  |  |
| b. | Abdominal pain |  |  |
| c. | Foul-smelling or pus vaginal discharge |  |  |
| d. | Vaginal bleeding (heavier than spotting) |  |  |
| e. | Pain passing urine |  |  |
| r. | Urinary frequency – passing urine more often |  |  |
| g. | Urinary urgency – need to pass urine quickly/difficulty in holding urine |  |  |
| h. | At the site of your caesarean section (cut/operation on your abdomen) |  |  |
|  | 1. Pus discharge |  |  |
|  | 1. Pain |  |  |
|  | 1. Swelling |  |  |
|  | 1. Redness |  |  |
|  | 1. Wound breakdown (wound edges separated) |  |  |
| i. | At the site of a perineal wound (cut or tear in the vagina) | Option of ‘no perineal wound’ |  |
|  | 1. Pus discharge |  |  |
|  | 1. Pain |  |  |
|  | 1. Swelling |  |  |
|  | 1. Wound breakdown (wound edges separated) |  |  |
| j. | Painful, red breast |  |  |
| k. | Swollen, hard area of the breast |  |  |
| l. | Productive cough (coughing up sputum) |  |  |
| m. | Difficulty breathing |  |  |

If No to Qu.1 and all of Qu.3 -> Qu. 7

1. How many days after giving birth did these symptoms start?
2. Where did you seek help for these problems? Tell me each place or person.
   1. Hospital where delivered
   2. A different hospital
   3. A lower level healthcare facility
   4. A private clinic
   5. A pharmacist/drug store
   6. A local shop (not a drug store)
   7. A traditional healer/doctor
   8. A family member
   9. A friend/neighbour
   10. Other – describe ___________________________
   11. Did not seek help - If current symptoms, advise to seek medical help
3. What diagnosis were you given? (Write ‘unknown’ if the woman does not know/remember. Write ‘no diagnosis’ if a diagnosis was not made e.g. if she only spoke to a friend or shop owner)

_____________________________________________________________

1. Have you taken any medicine in the last 3 weeks? (Mark any that apply and give name of drugs if known)
   1. Amoxil/amoxicillin
   2. Metronidazole
   3. Ampiclox
   4. Erythromycin
   5. Ciprofloxacin
   6. Alu/duocotexin/Mceto (oral antimalarial)
   7. Iv/im Artesunate/Artemether (antimalarial)
   8. Paracetamol
   9. Other
   10. Unknown treatment
   11. No treatment
2. Have you been readmitted to hospital in the last 3 weeks? Y/N If No, -> Qu.10
3. Was it the same hospital where you gave birth? Y/N
4. When were you readmitted (How many days after giving birth?)____
5. Are you currently breastfeeding your baby? Yes, exclusive/ Yes, mixed, /No

For the next questions, I would like you to say how difficult you find the following activities – not difficult, a little difficult or very difficult

1. Washing your whole body? Not at all/little/very
2. Taking care of your household responsibilities e.g. cleaning/cooking? No responsibilities/Not difficult/little/very
3. Picking up and carrying your baby? Not at all/little/very
4. Taking care of your baby e.g. washing them? Not at all/little/very

The next few questions ask about how you have been feeling in the last 7 days. These statements are about how you have felt in the past week (7 days), not just how you feel today. I will read the statements and give you a choice of responses.

In the last 7 days:

1. Have you looked forward to things with enjoyment?
   1. As much as I ever did (0)
   2. Rather less than I used to (1)
   3. Definitely less than I used to (2)
   4. Hardly at all (3)
2. Have you been so unhappy that you have had difficulty sleeping?
3. Yes, most of the time (3)
4. Yes, sometimes (2)
5. Not very often (1)
6. No, not at all (0)
7. Have you felt sad or miserable?
8. Yes, most of the time (3)
9. Yes, sometimes (2)
10. Not very often (1)
11. No, not at all (0)
12. Have you been so unhappy that you have been crying?
13. Yes, most of the time (3)
14. Yes, sometimes (2)
15. Not very often (1)
16. No, not at all (0)
17. Have thoughts of harming yourself occurred to you?
18. Yes, most of the time (3)
19. Yes, sometimes (2)
20. Not very often (1)
21. No, not at all (0)

Add up all the points for Qu. 13-17. Maximum score is 15.

If a woman scores 6 or more or has thoughts of harming herself, say to her, “there seem to be many things that are making you sad. This can be common for women who just gave birth. Would you like me to speak to your relative? Or would you like to speak to a social welfare officer?

**Newborn Questions (if baby was alive at the last phone survey). Otherwise go to Qu.10**

Now I will ask some questions about your baby:

1. Has your baby been unwell or suffered any problems/complications since we phoned you 3 weeks ago? Y/N – If No -> Qu.3
2. If Yes, Can you describe the problem and any diagnosis given.

______________________________________________________________________

NB If the baby has died, give condolences, then ask sensitively if the mother knows what her baby died from and if the baby died at home or in hospital. Free-text any information she provides. Offer your condolences again and ask her if she would like to speak to someone from the social welfare team. If so, offer to pass on her contact details to them. Go to Qu.10. Do not proceed with further questions about the baby. Text the contact details for the social welfare team to her after the interview

1. For each of the following symptoms, has your baby experienced it in the last 3 weeks, and are they experiencing it today?

|  | Symptom | Last 3 weeks Y/N | Today Y/N |
| --- | --- | --- | --- |
| a. | Fever |  |  |
| b. | Very cold (low temperature) |  |  |
| c. | Very fast breathing |  |  |
| d. | Chest indrawing (sucking in the ribs when breathing) |  |  |
| e. | Convulsions/fits |  |  |
| f. | Poor feeding/not feeding |  |  |
| g. | Only moving when stimulated |  |  |
| h. | Redness around the umbilical cord stump |  |  |
| i. | Pus discharge from the umbilical cord stump |  |  |

If No, to Qu.1 and Qu.3 -> Qu.6

1. How many days after birth did these symptoms start?
2. Where did you seek help for your baby? Tell me each place or person.
3. Hospital where delivered
4. A different hospital
5. A lower level healthcare facility
6. A private clinic
7. A pharmacist/drug store
8. A local shop (not a drug store)
9. A traditional healer/doctor
10. A family member
11. A friend/neighbour
12. Other – describe ___________________________
13. Did not seek help - If current symptoms, advise to seek medical help
14. What diagnosis was your baby given? Write ‘unknown’ if the woman does not know/remember. Write ‘no diagnosis’ if a diagnosis was not made e.g. if she only spoke to a friend or shop owner

_____________________________________________________________

1. Has your baby had any medicine in the last 3 weeks? (Mark any that apply and give name of drugs if known)
   1. Amoxil/amoxicillin
   2. Metronidazole
   3. Ampiclox
   4. Erythromycin
   5. Ciprofloxacin
   6. Alu/duocotexin/Mceto (oral antimalarial)
   7. Iv/im Artesunate/Artemether (antimalarial)
   8. Paracetamol
   9. Other
   10. Unknown treatment
   11. No treatment
2. Was your baby admitted to hospital in the last 3 weeks? Y/N If No, -> Qu.10
3. Was it the same hospital where your baby was born? Y/N
4. When were they admitted? (How many days since birth?)_____

If there is more than one baby (twins/triplets) then repeat all newborn questions

1. Thank you for your time.

This is the last time we will phone you as part of this study. Thank you very much for helping us. It is important that you attend your local health facility in 2 weeks’ time for your baby to receive their first immunisations.
