# Supplementary material for: Postnatal infection surveillance by telephone in Dar es Salaam, Tanzania: An observational cohort study
Source: PLoS One. 2021 Jul 1;16(7):e0254131. doi: 10.1371/journal.pone.0254131 (PMC8248639; doi:10.1371/journal.pone.0254131)
Supplement: S2 Appendix — (DOCX) [file pone.0254131.s002.docx]

**S2 Appendix: Hospital Case-note Extraction Form**

Patient ID Number:______________

Date of extraction:_________

Date of admission:___________

**Demographics**

Age of woman:

Referred from another health facility Y/N

Address:

**Pregnancy history**

Gravidity (number of pregnancies):

Parity (number of births at admission):

Gestational age at birth in weeks:

Number of babies in this pregnancy:

**Comorbidities**

Diabetes Yes/No/Unknown

Gestational Diabetes Yes/No/Unknown

Pre-eclampsia Yes/No/Unknown

Eclampsia Yes/No/Unknown

Pregnancy-induced hypertension Yes/No/Unknown

Antenatal haemorrhage Yes/No/Unknown

HIV positive Yes/No/Unknown

**Labour**

Premature rupture of membranes Y/N

Induction of labour Y/N

Artificial rupture of membranes Y/N

Augmentation of labour Y/N

IV line Y/N (look at observation chart)

**Delivery**

Date of delivery/birth:

Mode of delivery:

1. Spontaneous vertex delivery
2. Breach delivery
3. Vacuum extraction
4. Caesarean section

Episiotomy Y/N

Perineal tear Y/N

Perineal sutures Y/N

**Newborn outcomes**

Stillbirth Y/N If yes, skip to Infection questions

Apgar score at 5 minutes

Baby required suction Y/N

Baby required bag and mask Y/N

Baby admitted to neonatal ward Y/N

Baby with suspected sepsis

Baby received antibiotics Y/N

Baby alive at discharge Y/N

**Postpartum**

Postpartum haemorrhage Y/N

Mother’s temperature postpartum:

Mother alive at discharge Y/N

Date of discharge:_____

**Infection/Antibiotic use in mother**

Antibiotics received in labour Y/N.

If Yes, antibiotics given

1. Ampicillin
2. Ampiclox
3. Metronidazole
4. Ceftriaxone
5. Amoxicillin
6. Gentamicin
7. Erythromycin
8. Benzylpenicillin
9. Other__________________

Reason for antibiotics

1. Surgical prophylaxis – caesarean section
2. PROM
3. Manual removal of placenta
4. Perineal suture
5. Haemorrhage (APH/PPH)
6. Infection
7. Other:­­­­­­­­­­­­_____________
8. Unknown

Infection diagnosed:

1. Chorioamnionitis
2. Urinary tract infection
3. Respiratory tract infection
4. Sepsis
5. Other___________
6. Unknown

Antibiotics received after delivery Y/N.

If Yes, antibiotics given

1. Ampicillin
2. Ampiclox
3. Metronidazole
4. Ceftriaxone
5. Amoxicillin
6. Gentamicin
7. Erythromycin
8. Benzylpenicillin
9. Other_____________________

Reason for antibiotics

1. Surgical prophylaxis – caesarean section
2. PROM
3. Manual removal of placenta
4. Perineal suture
5. Haemorrhage (APH/PPH)
6. Infection
7. Other:­­­­­­­­­­­­_____________
8. Unknown

Infection diagnosed:

1. Chorioamnionitis
2. Urinary tract infection
3. Respiratory tract infection
4. Sepsis
5. Endometritis
6. Wound infection/SSI
7. Other___________
8. Unknown
