# Supplementary material for: Postnatal infection surveillance by telephone in Dar es Salaam, Tanzania: An observational cohort study
Source: PLoS One. 2021 Jul 1;16(7):e0254131. doi: 10.1371/journal.pone.0254131 (PMC8248639; doi:10.1371/journal.pone.0254131)
Supplement: S1 Table — (DOCX) [file pone.0254131.s003.docx]

**S1 Table: Demographic, pregnancy and newborn factors by delivery hospital for 840 women and 829 liveborn babies with hospital record data by study hospital**

|  | **Amana n(%) (N=425)** | **Temeke n(%) (N=394)** | **Total n(%)**  **(N=840)** |
| --- | --- | --- | --- |
| Median maternal age in years (IQR) | 25 (22-30) | 26 (23-31) | 25 (22-30) |
| Age grouped (years) |  |  |  |
| 18-24 | 184 (42.7) | 154 (37.7) | 338 (40.2) |
| 25-29 | 121 (28.1) | 114 (27.9) | 235 (28.0) |
| 30+ | 123 (28.5) | 126 (30.8) | 249 (29.6) |
| Missing | 3 (0.7) | 15 (3.7) | 18 (2.1) |
| Parity grouped |  |  |  |
| 0 | 161 (37.4) | 125 (30.6) | 286 (34.1) |
| 1 | 133 (30.9) | 122 (29.8) | 255 (30.4) |
| 2 | 61 (14.2) | 87 (21.3) | 148 (17.6) |
| 3+ | 67 (15.6) | 58 (14.2) | 125 (14.9) |
| Missing | 9 (2.1) | 17 (4.2) | 26 (3.1) |
| Preterm birth (<37 weeks gestation) | 37 (8.6) | 44 (10.8) | 81 (9.6) |
| Missing | 184 (42.7) | 145 (35.5) | 329 (39.2) |
| Referred in | 17 (3.9) | 5 (1.2) | 22 (2.6) |
| Missing | 1 (0.2) | 0 | 1 (0.1) |
| Diabetes/GDM | 0 | 2 (0.5) | 2 (0.2) |
| Missing | 0 | 1 (0.2) | 1 (0.1) |
| Hypertensive disorders | 17 (3.9) | 17 (4.2) | 34 (4.1) |
| Missing | 1 (0.2) | 5 (1.2) | 6 (0.7) |
| HIV | 22 (5.1) | 15 (3.7) | 37 (4.4) |
| Missing/not available | 8 (1.9) | 7 (1.7) | 15 (1.8) |
| PROM | 18 (4.2) | 11 (2.7) | 29 (3.5) |
| Missing | 1 (0.2) | 2 (0.5) | 3 (0.4) |
| Induction of labour | 18 (4.2) | 2 (0.5) | 20 (2.4) |
| Missing | 1 (0.2) | 1 (0.2) | 2 (0.2) |
| Artificial rupture of membranes | 12 (2.8) | 2 (0.5) | 14 (1.7) |
| Missing | 0 | 2 (0.5) | 2 (0.2) |
| Augmentation of labour | 22 (5.1) | 2 (0.5) | 24 (2.9) |
| Missing | 0 | 3 (0.7) | 3 (0.4) |
| Episiotomy | 2 (0.5) | 8 (2.0) | 10 (1.2) |
| Missing | 11 (2.6) | 3 (0.7) | 14 (1.7) |
| Perineal tear | 168 (39.0) | 82 (20.1) | 250 (29.8) |
| Missing | 1 (0.2) | 2 (0.5) | 3 (0.4) |
| Perineal suture (N=260 women with perineal trauma) | 166/170 (97.7) | 88/90 (97.8) | 254 (97.7) |
| Missing | 0 | 1 (1.1) | 1 (0.4) |
| PPH | 2 (0.5) | 7 (1.7) | 9 (1.1) |
| Missing | 1 (0.2) | 1 (0.2) | 2 (0.2) |
| Antibiotics in labour | 53 (12.3) | 9 (2.2) | 62 (7.4) |
| Missing | 3 (0.7) | 3 (0.7) | 6 (0.7) |
| Antibiotics postpartum | 425 (98.6) | 96 (23.5) | 521 (62.0) |
| Missing | 1 (0.2) | 7 (1.7) | 8 (1.0) |
| **Newborn Factors** |  |  | **Total (N=829)** |
| Apgar Score at 5 minutes <7 | 5 (1.20) | 5 (1.2) | 10 (1.2) |
| Missing | 2 (0.5) | 1 (0.2) | 3 (0.4) |
| Bag and mask | 12 (2.9) | 5 (1.2) | 17 (2.1) |
| Missing | 0 | 4 (1.1) | 4 (0.5) |
| Admission | 21 (5.0) | 1 (0.2) | 22 (2.7) |
| Missing | 0 | 2 (0.5) | 2 (0.2) |
