# Supplementary material for: Postnatal infection surveillance by telephone in Dar es Salaam, Tanzania: An observational cohort study
Source: PLoS One. 2021 Jul 1;16(7):e0254131. doi: 10.1371/journal.pone.0254131 (PMC8248639; doi:10.1371/journal.pone.0254131)
Supplement: S2 Table — (DOCX) [file pone.0254131.s004.docx]

**S2 Table: Associations between potential risk factors and possible maternal postnatal infection**

| **Factor** | **Total women** | **Episodes of postnatal infection** | **Person-time (months)** | **Rate of infection per 1000 person months** | **Crude Rate ratio (95% CI)**  **N=754^a^** | **Wald**  **p-value** |
| --- | --- | --- | --- | --- | --- | --- |
| All women | 791 | 56 | 705.3 | 79.4 (61.1-103.2) |  |  |
| Delivery mode |  |  |  |  |  |  |
| Vaginal | 645 | 39 | 578.1 | 67.5 | 1 | 0.02 |
| Caesarean section | 146 | 17 | 127.3 | 133.6 | 1.95 (1.12-3.37) |  |
| Maternal age (years) |  |  |  |  |  |  |
| 18-24 | 303 | 15 | 167.9 | 56.0 | 1 | 0.05 |
| 25-29 | 212 | 23 | 186.0 | 123.6 | 2.20 (1.15-4.28) |  |
| 30+ | 223 | 16 | 204.3 | 78.3 | 1.43 (0.72-2.84) |  |
| Hospital |  |  |  |  |  |  |
| Amana | 403 | 28 | 362.0 | 77.4 | 1 | 0.87 |
| Temeke | 388 | 28 | 343.4 | 81.5 | 1.04 (0.62-1.75) |  |
| Parity |  |  |  |  |  |  |
| 0 | 252 | 15 | 224.8 | 66.7 | 1 | 0.81 |
| 1 | 233 | 19 | 206.9 | 91.8 | 1.33 (0.69-2.56) |  |
| 2 | 131 | 11 | 115.0 | 95.7 | 1.37 (0.65-2.89) |  |
| 3+ | 113 | 8 | 103.6 | 77.2 | 1.16 (0.48-2.81) |  |
| Preterm birth (<37 weeks) |  |  |  |  |  |  |
| No | 392 | 30 | 346.5 | 86.6 | 1 | 0.89 |
| Yes | 69 | 5 | 62.5 | 80.1 | 0.94 (0.37-2.35) |  |
| HIV infection |  |  |  |  |  |  |
| No | 705 | 53 | 630.0 | 84.1 | 1 | 0.38 |
| Yes | 34 | 1 | 28.2 | 35.4 | 0.41 (0.06-2.91) |  |
| Hypertensive disorders |  |  |  |  |  |  |
| No | 721 | 52 | 642.8 | 80.9 | 1 | 0.96 |
| Yes | 28 | 2 | 24.5 | 81.5 | 0.96 (0.25-3.75) |  |
| PROM |  |  |  |  |  |  |
| No | 727 | 55 | 648.5 | 84.8 |  |  |
| Yes | 24 | 0 | 21.6 | 0.00 |  |  |
| ARM |  |  |  |  |  |  |
| No | 738 | 55 | 657.6 | 83.6 |  |  |
| Yes | 14 | 0 | 13.3 | 0.00 |  |  |
| PPH |  |  |  |  |  |  |
| No | 746 | 53 | 665.2 | 79.7 | 1 | 0.44 |
| Yes | 7 | 1 | 6.0 | 166.3 | 2.10 (0.33-13.49) |  |
| Antibiotics in labour |  |  |  |  |  |  |
| No | 697 | 48 | 622.6 | 77.1 | 1 | 0.17 |
| Yes | 51 | 6 | 43.7 | 137.3 | 1.75 (0.78-3.91) |  |
| Postpartum antibiotics |  |  |  |  |  |  |
| No | 277 | 18 | 246.4 | 73.0 | 1 | 0.49 |
| Yes | 469 | 37 | 417.9 | 88.5 | 1.22 (0.69-2.16) |  |

^a^Values imputed for variables with missing data except for preterm birth where a large amount of data was missing.
