# Supplementary material for: Postnatal infection surveillance by telephone in Dar es Salaam, Tanzania: An observational cohort study
Source: PLoS One. 2021 Jul 1;16(7):e0254131. doi: 10.1371/journal.pone.0254131 (PMC8248639; doi:10.1371/journal.pone.0254131)
Supplement: S3 Table — (DOCX) [file pone.0254131.s005.docx]

**S3 Table: Associations between potential risk factors and possible newborn infection**

| **Factor** | **Total newborns** | **Episodes of possible infection** | **Person-time (months)** | **Rate of infection per 1000 person months** | **Crude rate ratio (95% CI)**  **N=725^a^** | **Wald p-value** |
| --- | --- | --- | --- | --- | --- | --- |
| All babies | 762 | 82 | 677.4 | 121.1 (97.5-150.3) |  |  |
| Resuscitation (bag and mask) |  |  |  |  |  |  |
| No | 709 | 75 | 629.9 | 119.1 | 1 | <0.001 |
| Yes | 11 | 5 | 8.7 | 574.3 | 4.61 (2.35-9.04) |  |
| Antibiotics in labour |  |  |  |  |  |  |
| No | 674 | 69 | 598.5 | 115.3 | 1 | 0.01 |
| Yes | 47 | 10 | 39.9 | 250.9 | 2.15 (1.18-3.91) |  |
| Delivery mode |  |  |  |  |  |  |
| Vaginal | 621 | 64 | 552.2 | 115.9 | 1 | 0.35 |
| Caesarean section | 141 | 18 | 125.1 | 143.8 | 1.24 (0.74-2.09) |  |
| PROM |  |  |  |  |  |  |
| No | 698 | 75 | 617.7 | 121.4 | 1 | 0.37 |
| Yes | 24 | 4 | 22.1 | 180.6 | 1.53 (0.61-3.84) |  |
| Maternal age (years) |  |  |  |  |  |  |
| 18-24 | 291 | 29 | 256.9 | 112.9 | 1 | 0.51 |
| 25-29 | 203 | 27 | 280.4 | 149.6 | 1.34 (0.79-2.28) |  |
| 30+ | 216 | 22 | 193.0 | 114.0 | 1.05  (0.60-1.84) |  |
| Hospital |  |  |  |  |  |  |
| Amana | 388 | 41 | 347.1 | 118.1 | 1 | 0.94 |
| Temeke | 374 | 41 | 330.3 | 124.1 | 1.04 (0.67-1.61) |  |
| Preterm (<37 weeks gestation) |  |  |  |  |  |  |
| No | 376 | 38 | 330.6 | 114.9 | 1 | 0.65 |
| Yes | 67 | 8 | 59.5 | 134.5 | 1.18 (0.57-2.44) |  |
| Postpartum antibiotics |  |  |  |  |  |  |
| No | 266 | 21 | 236.8 | 88.7 | 1 | 0.07 |
| Yes | 452 | 58 | 399.5 | 145.2 | 1.59 (0.96-2.62) |  |
| HIV infection |  |  |  |  |  |  |
| No | 677 | 78 | 602.8 | 129.4 | 1 | 0.41 |
| Yes | 33 | 2 | 26.0 | 77.1 | 0.56 (0.14-2.20) |  |
| Hypertensive disorders |  |  |  |  |  |  |
| No | 696 | 77 | 617.3 | 124.7 | 1 | 0.68 |
| Yes | 24 | 2 | 21.0 | 95.1 | 0.75 (0.19-2.94) |  |
| Artificial rupture of membranes |  |  |  |  |  |  |
| No | 709 | 80 | 628.2 | 127.4 |  |  |
| Yes | 14 | 0 | 12.6 | 0 |  |  |
| Postpartum haemorrhage |  |  |  |  |  |  |
| No | 717 | 78 | 635.9 | 122.7 | 1 | 0.82 |
| Yes | 7 | 1 | 6.7 | 149.2 | 1.24 (0.20-7.76) |  |

^a^values imputed for variables with missing data except for preterm birth which had a large amount of missing data
