# Supplementary material for: Postnatal infection surveillance by telephone in Dar es Salaam, Tanzania: An observational cohort study
Source: PLoS One. 2021 Jul 1;16(7):e0254131. doi: 10.1371/journal.pone.0254131 (PMC8248639; doi:10.1371/journal.pone.0254131)
Supplement: Attachment — Submitted filename: Response to reviewers.docx [file pone.0254131.s006.docx]

Dear Editors and reviewers,

Many thanks for your helpful comments. Please find our responses in the table below.

| **Editor’s comments** |  |
| --- | --- |
| Thank you for stating the following in the Competing Interests section:  "I have read the journal's policy and the authors of this manuscript have the following competing interests: SLW, AMK, AK, WM and WJG received personal salary funded by The Soapbox Collaborative for their work on the study. SLW received a Research Degree Travel Grant from LSHTM. Other authors declare no competing interests"  Please confirm that this does not alter your adherence to all PLOS ONE policies on sharing data and materials | The competing interests statement is updated as follows:  "I have read the journal's policy and the authors of this manuscript have the following competing interests: SLW, AMK, AK, WM and WJG received personal salary funded by The Soapbox Collaborative for their work on the study. SLW received a Research Degree Travel Grant from LSHTM. AMR received salary from the UK Medical Research Council and the UK Department for International Development (DFID) under the MRC/DFID Concordat agreement. Other authors declare no competing interests. This does not alter our adherence to PLOS ONE policies on sharing data and materials." |
| Footnote ‘b’ in tables 5 and 6 is not clear. Moreover, I could not identify footnote 'b' in Table 6. | Thank you for asking for this clarification. We have now combined the information into a single footnote in both tables:  Values imputed for variables with missing data, except for Preterm birth where the amount of missing data was considered too large to impute  We hope it is now clear that imputation was carried out for all variables except Preterm birth |
| Please use a consistent style for footnotes. In table 2, a special character is used but elsewhere letters are used. | Apologies for this error, we now use a letter in table 2 as well |
| RR can mean Risk ratio or rate ratio. Please specify. Given that you used Cox regression, why not report the effects as hazard ratios. | As suggested, we have changed the reporting to hazard ratios |
| In reporting the results of association, especially in the abstract, please state explicitly the comparison group. | Thank you for this request. We have now explicitly stated the comparison group for each association, in both the abstract, and the results section |
| **Journal comments** |  |
| . Please ensure that your manuscript meets PLOS ONE's style requirements, including those for file naming. The PLOS ONE style templates can be found below | The headings and supplementary file names have been updated as specified. |
| We note that the grant information you provided in the ‘Funding Information’ and ‘Financial Disclosure’ sections do not match.  When you resubmit, please ensure that you provide the correct grant numbers for the awards you received for your study in the ‘Funding Information’ section. | We apologise for this. The Funding Information now includes the MRC/DfID grant (and grant number) for AMR.  The Soapbox Collaborative is a charity and does not provide grant numbers.  We hope all the expected information is now included. |
| We note that you have stated that you will provide repository information for your data at acceptance. Should your manuscript be accepted for publication, we will hold it until you provide the relevant accession numbers or DOIs necessary to access your data. If you wish to make changes to your Data Availability statement, please describe these changes in your cover letter and we will update your Data Availability statement to reflect the information you provide. | As described in our cover letter, we need to change our data availability statement. Following Tanzanian guidance, as reflected in the project Data Transfer Agreement, any third party wishing to access the data will need to request written consent from Ifakara Health Institute, who own the data. The data can be accessed from the IHI repository https://data.ihi.or.tz/index.php/catalog/edit/1. Anyone requesting the data will be required to submit a brief abstract narrating the reasons for requesting access to the data. The system will send an email to the data team and the local PI. |
| **Reviewer #1** |  |
| This study estimates rates of postnatal infection among women and infants in Dar es Salaam, Tanzania, using telephone surveillance. It demonstrates the feasibility of telephone surveillance for this purpose in Tanzania, and provides the first post-discharge estimates of maternal and newborn infection rates in this setting, as well as associated risk factors. It is clear, well-written, the methods and conclusions are sound, and it is a valuable addition to the literature on maternal and newborn health in sub-Saharan Africa. | Thank you very much for these kind and positive comments |
| Line 72: Can any more information about the selected hospitals be provided? Are they public/private? What is their catchment area? How many hospitals of this size are there in Dar es Salaam? | The hospitals are two of the three public regional referral hospitals in Dar es Salaam, covering the districts of Ilala and Temeke. This has been reflected in the text.  For information, there are 28 hospitals at this level in Tanzania. |
| Line 92: Given that one of the major limitations of the study is around the ability of the questionnaire to accurately identify infections, could you provide more details here on how the standard definitions were adapted? Are there any existing questionnaires for measuring postnatal infection that were considered but not used? If so, it would be helpful to discuss this here too. | In essence, we adapted the definitions by removing all laboratory investigations and any clinical signs that could not be easily reported by the women, for example, temperature readings, abdominal tenderness, perineal wound redness. We were less rigid with some definitions e.g. superficial wound infection, to allow for these necessary omissions. A more detailed account is being written up elsewhere. A short sentence has been added to the manuscript.  These definitions were adapted by the first author to include only symptoms and signs easily reported by the women.  We did not search extensively for other questionnaires, but from our work in this field, including a systematic review of maternal peripartum infection, we are not aware of any published, (validated) telephone questionnaires for this purpose. |
| Line 128: What was the frequency of discordant data, stillbirths and twins/triplets? | Among the 12 interviews with mothers of liveborn twins, only six record data on the second twin, partially due to a fault in the ODK programming.  Among mothers with hospital and telephone data, 28 have a baby who dies in the first 7 days. Of these, maternal cause-of-death reports are consistent with 7/8 of the hospital-recorded stillbirths. However, the hospital records miss 4 more stillbirths reported by mothers and at Temeke hospital no stillbirths are recorded, which we consider highly unlikely. |
| Reviewer #2 |  |
| Line 203 - 28 early stillbirths occurred, did you include these in your final analysis? | Women giving birth in hospital and recorded in the delivery register were eligible for recruitment, including those with stillbirths. All recruited women who were reached for interview were included in the analysis of maternal postnatal infection.  Stillbirths contribute to the 28 deaths occurring up to day 7. Stillbirths were not included in the analysis of newborn infection |
| Line 204 - between day 8-28 additional 6 babies died, did you include these numbers in you final analysis? | As described in the methods, line 104, babies who died after day 7 contributed to the analysis (infection rate and analysis of associations) up to day 7 only. |
| Line 217 - What do you mean by "weak evidence" of higher infection rates in women 25-29 years ( you make reference to table 5: [2.14 (1.12- 4.09): p=0.06]. How do you explain these p-value versus CI above 1.0? | Thank you for noticing this. The p-value refers to the overall association between the three categories of age-group and infection rate rather than the 25-29 year group. We have rephrased the text to better reflect the result.  There was also weak evidence of an association between women’s age-group and infection (p=0.06) with the highest infection rates occurring in women aged 25–29. |
| Line 235: Maternal postnatal infection was associated with increased odds of possible newborn infection (crude Odds Ratio 2.74, 95%CI 1.16–6.48, p=0.02). Author should rephrase this statement or provide some explanation as well as discuss this in the section for discussion of results | The statement has been rephrased as follows:  Mothers with postnatal infection in the first 7 days had an increased risk of their baby suffering possible newborn infection during this time period, compared to mother’s without infection  This finding is currently discussed on line 297:  There was some evidence of a crude association between maternal and newborn infection, also found in a systematic review of maternal infection in labour, suggesting a shared aetiology for some infections and highlighting the importance of caring for woman and newborn synergistically. |
| Line 238-239: Please rephrase to provide easy readability | We hope the following revision of this sentence is clearer to read  At the day-7 interview, 5/43 (12%) women with possible postnatal infection reported they had been readmitted to hospital as compared with only 5/696 (0.7%) women without infection. |
